# Kidney fibrosis: from mechanisms to therapeutic medicines

**DOI:** 10.1038/s41392-023-01379-7

**Published:** 2023-03-17

**Authors:** Rongshuang Huang, Ping Fu, Liang Ma

**Affiliations:** Kidney Research Institute, Division of Nephrology, West China Hospital, Sichuan University, Chengdu, 610041 China

**Keywords:** Kidney diseases, Kidney diseases

## Abstract

Chronic kidney disease (CKD) is estimated to affect 10–14% of global population. Kidney fibrosis, characterized by excessive extracellular matrix deposition leading to scarring, is a hallmark manifestation in different progressive CKD; However, at present no antifibrotic therapies against CKD exist. Kidney fibrosis is identified by tubule atrophy, interstitial chronic inflammation and fibrogenesis, glomerulosclerosis, and vascular rarefaction. Fibrotic niche, where organ fibrosis initiates, is a complex interplay between injured parenchyma (like tubular cells) and multiple non-parenchymal cell lineages (immune and mesenchymal cells) located spatially within scarring areas. Although the mechanisms of kidney fibrosis are complicated due to the kinds of cells involved, with the help of single-cell technology, many key questions have been explored, such as what kind of renal tubules are profibrotic, where myofibroblasts originate, which immune cells are involved, and how cells communicate with each other. In addition, genetics and epigenetics are deeper mechanisms that regulate kidney fibrosis. And the reversible nature of epigenetic changes including DNA methylation, RNA interference, and chromatin remodeling, gives an opportunity to stop or reverse kidney fibrosis by therapeutic strategies. More marketed (e.g., RAS blockage, SGLT2 inhibitors) have been developed to delay CKD progression in recent years. Furthermore, a better understanding of renal fibrosis is also favored to discover biomarkers of fibrotic injury. In the review, we update recent advances in the mechanism of renal fibrosis and summarize novel biomarkers and antifibrotic treatment for CKD.

## Introduction

Chronic kidney disease (CKD) is defined as abnormalities of renal structure or function, present for >3 months, with implications for health.^1^ The most commonly used diagnostic criteria in clinics are estimated glomerular filtration rate (eGFR) <60 mL/min/1.73 m^2^ or urinary albumin-to-creatinine ratio (ACR) ≥ 30 mg/g. Ascertaining the actual prevalence of CKD is a difficult task owing to its early-stage asymptomatic nature, but the disease is predicted to affect 10–14% of global population.^2–4^ Regardless of diverse causes, CKD is featured by progressive and irreversible nephron loss, microvascular damage, decreased regenerative capacity, inflammation, oxidative stress, and metabolic changes, which ultimately led to renal failure and end-stage kidney disease.^5^ The impact of CKD on worldwide morbidity and mortality is rapidly increasing,^6,7^ indicating the shortcoming of therapeutic drugs at present.

Kidney fibrosis is the common pathological feature and final manifestation of CKD, whose morphological characteristics include glomerulosclerosis, tubule atrophy, interstitial chronic inflammation, and fibrogenesis, as well as vascular rarefaction.^8^ Fibrosis occurs when wound healing is deregulated, leading to excessive extracellular matrix (ECM) protein accumulation such as fibronectin and collagens.^9^ When kidneys are injured, local fibroblasts and pericytes are activated, increasing their contractility, secreting inflammatory mediators, and synthesizing ECM components, which trigger wound healing. However, if the damage is repetitious or severe, the ECM proteins persistently accumulate in the kidneys, resulting in tissue disruption, renal dysfunction, and ultimately organ failure.^10^ Despite substantial progress in understanding kidney fibrotic mechanism, there remains a translational barrier from the identification of the promising antifibrotic therapeutic drug target to the transformation of this knowledge into clinical application for human health.

The kidney organ consists of some anatomically and functionally discrete segments, where diverse cell types with sophisticated mechanisms participate in the occurrence and progression of renal fibrosis.^11^ In particular, the rapidly developing technologies of spatial and single-cell transcriptomics could facilitate dissecting genetic programming, signal pathway, and the mechanism of cell crosstalk that underlie kidney function in physiological conditions, as well as the dysfunctions in fibrotic condition.^12^

Epigenetics controls gene expression without altering DNA sequence and affect the gene and environment crosstalk.^13^ Epigenetic regulation exerted fundamental and crucial functions in cell biology of kidneys through the action of DNA methylation, chromatin modifications via epigenetic factors and interaction with transcription factors, and non-coding RNAs.^14^ New findings in epigenetics can drive the development of not only the mechanism of kidney fibrosis, but also biomarkers and targeted drugs for the CKD’s diagnosis, prognosis, and therapy.

In the review, we updated recent advances in the mechanism of kidney fibrosis from new perspectives and summarize the new and promising biomarker and antifibrotic treatment for patients with fibrotic kidney diseases.

## Fibrotic niche in kidney fibrosis

**Fig. 1:**
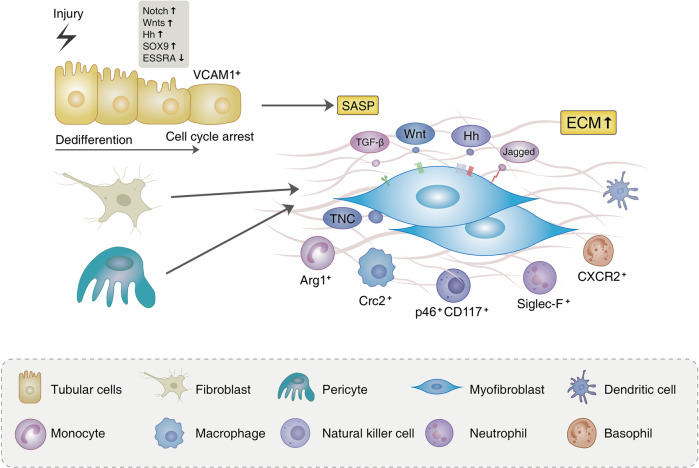
Origination and activation of myofibroblasts. In tubulointerstitium, injury results in epithelial dedifferentiation, which is characterized by the upregulation of Notch, Wnt, Hedgehog (Hh), and SOX9 pathways. Persistent damage leads to cycle arrest and senescence of tubular epithelial cells, accompanying the secretion of profibrotic factors and senescence-associated secretory phenotype (SASP). Injured VCAM-1^+^ tubules secrete paracrine mediators such as TGF-β, Hh, and Wnt ligands, which impact interstitial pericytes and fibroblasts to activate myofibroblast differentiation, proliferation, and ECM accumulation. Of note, the different population of immune cell including macrophage, lymphocyte, neutrophil, and basophil also have been found in the interstitial space. And these cells expressing specific markers play an important part in kidney fibrosis

Increasing evidence has supported the idea that organ fibrosis starts from ‘fibrotic niche’—a complex interplay between the injured parenchyma and multiple non-parenchymal cell lineages spatially located within areas of scarring.^15–17^ Kidney spatial transcriptomic analysis demonstrated that mesenchymal cells, immune cells, and specific types of tubular epithelial cells were the cellular components of fibrotic niche within the human kidney.^18^ Although the main cell types of fibrotic niche have been identified, the functional heterogeneity and the interaction of cell lineages need further clarifications (Fig. 1).

### Myofibroblast

Myofibroblasts activation and subsequent ECM accumulation are major events in kidney fibrosis. The activated myofibroblast works as the prominent contributor to renal fibrosis due to its ability to produce the most matrix.^19^ The alpha-smooth-muscle actin (α-SMA) is a specific marker that activates fibroblasts into myofibroblasts. Likewise, other interstitial cells such as pericyte and vascular smooth-muscle cells also express α-SMA. To distinguish myofibroblasts, vimentin, collagen-1α1 (Col1a1), CD73, platelet-derived growth factor receptor beta (PDGFRβ), and fibroblast-specific protein-1 (FSP1)/S100A4 could be used.^10,12^ The number of myofibroblast is rare in normal condition, but increases sharply in fibrotic kidneys. However, the origin of myofibroblast remains controversial. Resident fibroblasts,^20,21^ pericytes,^21,22^ mesenchymal stem cell (MSC)-like cells,^23^ epithelial cells,^24,25^ endothelium,^26,27^ and circulating bone marrow-derived cells are all candidates for possible precursors.^28–30^ Recently, a published single-cell atlas of CKD in human could go a long way toward answering this question. Christoph et al. totally profiled 87362 kidney cortex cells from 13 patients of CKD owing to hypertensive nephrosclerosis.^18^ After defining myofibroblasts that express a large proportion of ECM genes, they reported three main myofibroblast sources in kidneys: PDGFRα^+^PDGFRβ^+^MEG3^+^ fibroblast; PDGFRβ^+^COLEC11^+^CXCL12^+^ fibroblast; and PDGFRα^–^PDGFRβ^+^RGS5^+^NOTCH3^+^ pericytes. And the identified chemokine CXCL12-expressing cells are exactly similar to the Gli1^+^ MSC-like cells.^23^ Cessation of cell cycle characterized the pericyte- or fibroblast-to-myofibroblast differentiation. The early activating protein-1 (AP-1) signaling and late TGF-β signaling played regulatory roles in the differentiation of fibroblasts and pericytes into myofibroblasts. Another study that conducted a combination of ATAC-seq (a method detecting chromatin accessibility) with single-cell sequencing identified transcription factor Runx1 was a direct driver of human kidney fibroblasts transdifferentiating into myofibroblast, and abnormal Runx1 triggered the expression of several myofibroblast genes (Fn1, Col13A1, Tgfbr1, Twist2, and Postn).^31^ In addition, the increase of ECM genes was inapparent in tubular epithelial cells and suggested that the long-debated epithelial-to-mesenchymal transition (EMT) exerted a minor contribution to kidney fibrosis.^18^

The mechanism of myofibroblast activation is the central issue in kidney fibrosis. As above-mentioned, a fibrotic niche forms after injury, where the injured tubular cells and the infiltrated immune inflammatory cells could secrete various profibrotic mediators, which target myofibroblast precursors via autocrine or paracrine pathway, leading to the activation of myofibroblast.^16^ The sources of profibrotic factors (Table 1) and key signals that mediate myofibroblast activation are introduced in the subsequent part.

**Table 1:** Tubule-derived factors regulating myofibroblast activation

Profibrotic factor	Main effects	Ref.
TGF-β	TGF-β (1) directly triggers ECM synthesis via Smad3-dependent or independent manners, (2) suppresses the ECM degradation by inhibiting MMPs and inducing natural inhibitors of MMPs (like TIMPs), (3) induces trans-differentiation of myofibroblasts, and (4) mesangial cells proliferation and elimination of TECs, podocytes, and endothelial cells	^22–31,273–276^
HER2	Tubule-derived HER2 induces the profibrotic CTGF expression via activating STAT3	^277^
FGF2	Autophagy-mediated expression and secretion of FGF2 activates fibroblasts	^278,279^
Wnt	Epithelial-derived Wnt ligands induce fibroblasts proliferation and differentiation into myofibroblasts	^68,69^
Hh	Hh promotes the transcription factor Glis nuclear translocation, and Shh upregulates the expression of Snail, α-SMA, fibronectin, and collagen I in fibroblasts	^75,280^
PDGFs	PDGFs mediate the proliferation, migration, and activation of mesangial cells, fibroblasts, and vascular smooth-muscle cells	^22,281^
CTGF	CTGF (1) is a co-factor for TGF-β, (2) activates the Wnt signaling pathway via interacting with LRP6, and (3) induces the trans-differentiation of pericytes and fibroblasts into myofibroblasts	^282–284^
TIMP	Timp1 promotes fibroblast proliferation	^285^
Lcn2	Lcn2 increases collagen production in tubular cells	^286^
INHBB	INHBB promotes interstitial fibroblast activation through the release of profibrotic activin B from the injured TECs	^83^
Connexin 43	Cx43 mediates the ATP release from TECs, inducing the release of CXCL10 from peritubular macrophage and activation of intrarenal fibroblasts	^287^
DACH1	DACH1 controls the expression of cell cycle and myeloid chemotactic factors (e.g., CSF1, CCL2), contributing to macrophage infiltration and fibrosis development.	^288^

*α-SMA* alpha-smooth-muscle actin, *ATP* adenosine triphosphate, *CCL2* chemokine (C-C motif) ligand 2, *CSF1* colony-stimulating factor 1, *CTGF* connective tissue growth factor, *CXCL10* C-X-C motif chemokine ligand 10, *Cx43* Connexin 43, *DACH1* Dachshund homolog 1, *ECM* extracellular matrix, *FGF2* fibroblast growth factor 2, *HER2* human epidermal growth factor receptor 2, *Hh* hedgehog, *INHBB* inhibin subunit beta B, *Lcn2* lipocalin, *LRP6* low-density lipoprotein receptor-related protein 6, *MMP* matrix metallopeptidase, *PDGF* platelet-derived growth factor, *Shh* Sonic hedgehog, *STAT3* signal transducer and activator of transcription 3, *TEC* tubular epithelial cell, *TGF-β* transforming growth factor-b, *TIMP* tissue inhibitor of matrix metalloproteinase 1

There is a growing recognition that certain markers of myofibroblasts could be employed as therapeutic targets. Christoph et al. found that naked cuticle homolog 2 (NKD2) was especially expressed in terminally differentiated human and mouse myofibroblasts.^18^ Activity of TGF-β, Wnt, and tumor necrosis factor (TNF) signal pathway increased in NKD2^+^ myofibroblasts rather than NKD2^−^ cells. And NKD2 gene knockout cells showed the loss of ECM modulators, collagens, and glycoproteins. Therefore, the NKD2-marked myofibroblasts in fibrotic kidneys are essential for the expression of collagen and represent a promising drug target. Although Gli1^+^ MSC-like cells is the main source of myofibroblast and could be therapeutically targeted, the absence of Gli1^+^ MSCs alone is also able to trigger capillary rarefaction,^32^ which means the direct inhibition of Gli1^+^ cells may cause hypoxia-induced kidney fibrosis.^33^ Liang et al. have shown that conditional knockout of Yap/Taz in Gli1^+^ cells of mice retarded unilateral ureteral occlusion (UUO)-induced myofibroblast accumulation, ECM deposition, and tubulointerstitial fibrosis.^34^ Thus, the precise regulation of Gli1^+^ cells is required to treat or delay fibrosis. Myofibroblasts have other specific markers, such as integrin αvβ3,^35^ fibronectin,^36^ and CD248,^37^ which have been used to design targeted drug delivery systems to treat kidney fibrosis. However, the development of effective antifibrotic drugs remains challenging.

### Tubules and tubulointerstitial crosstalk

The tubules make up the majority of kidney organ and are sensitive to various injuries such as ischemia, hypoxia, toxins, proteinuria, and metabolic disturbance. In CKD, the injured epithelium is now recognized as both victims and contributors to the progression of fibrotic kidney diseases.^38^ Although tubular epithelial-to-mesenchymal transition provided a minor contribution to the myofibroblast pool, tubulointerstitial crosstalk initiated by injured tubules is a core driver of CKD progression. As acknowledged, tubular epitheliums possess the formidable capability of self-repair.^39^ However, when the insult is repetitive and ongoing, injured tubules fail to re-differentiate after dedifferentiation,^40^ which produce and release bioactive molecules to recruit inflammatory cells and activate myofibroblast differentiation, proliferation, and matrix secretion.^41^ The cytokines, growth factors, and key mediators in kidneys produced by epitheliums are listed in Table 1.

#### Profibrotic features of injured tubules

In addition to the above-mentioned characteristics, single-cell RNA transcriptomics (scRNA-seq) is beneficial to the precise identification of tubular cells with profibrotic characteristics. A population of dedifferentiated VCAM-1^+^ proximal tubule cells has been shown to exert broad relevance in fibrotic kidneys.^42,43^ A recent human kidney cell atlas revealed that adaptive and/or maladaptive tubule epithelial cells in proximal tubule (PT) and thick ascending limb (TAL) shared the common expression of prominin 1 (PROM1), hepatitis A virus cellular receptor 1 (HAVCR1), doublecortin domain containing protein-2 (DCDC2), and occupied a core location in fibrotic niche via multiple cell-cell interactions, thus inducing ECM deposition, myofibroblast differentiation, and inflammation.^44^ Another mouse single-cell transcriptomic analysis found that transient inflammatory proximal tubule cell state appeared after slight kidney damage, with the significant downregulation of glutathione metabolism-related genes which triggering these cells sensitive to ferroptotic stress.^45^ This study indicates that ferroptotic stress recruits proinflammatory cells as key participant in maladaptive repair.

#### Signal pathways in tubular differentiation

Notch, Wnt, and Hedgehog have been recognized as key developmental signaling pathways closely related to cell differentiation. The transient activation of the three signal pathways is required for injured tissue/organ repair, but sustained activation is believed to aggravate fibrotic progression. Evidence from single-cell transcriptomic data verify their functions and roles in renal fibrosis.

##### Notch

Activation of Notch signaling scarcely appears in adult kidney, and its re-expression in injured and fibrotic kidney is related to regeneration and repair.^46^ Although the proliferation of undifferentiated cells seems to be crucial for the replacement of lost tubular cells, on the other hand, the impeding effect of Notch signaling on differentiation impairs kidney function with a high probability.^47^ In the kidneys of CKD patients and mice, the Notch signaling is reinduced during fibrosis. In mouse experimental models, inducing the cleaved Notch1 expression caused tubular dilatation and atrophy, accompanied by matrix accumulation, myofibroblast activation, and immune cell infiltration in interstitium.^46^ In contrast, genetic or pharmacological inhibition of Notch signaling significantly reduced renal tubulointerstitial fibrosis.^25,48,49^ Moreover, as a key crosstalk signal pathway, Notch signaling is a major component of tubule-interstitial communication by paracrine effects. Increased tubular epithelial Notch expression was related with an elevated TGF-β level, which directly activates myofibroblasts.^50,51^ Snai1, one of the downstream genes of Notch, was once regarded as a main regulator of the EMT program.^52^ However, the linage-tracing study did not advocate the effects of epithelial-to-mesenchymal transition in renal fibrosis.^18,53^ Later, partial EMT has been proposed, which indicated that dedifferentiated tubules do not fully translate into myofibroblasts, but exacerbated kidney fibrosis via the secretion of mediators to trigger the differentiation of myofibroblast.^52,54^

##### Wnt

Wnt is comparatively invisible in normal condition of adult kidneys, but is initiated in human kidney diseases.^55^ Activation of Wnt after acute kidney injury (AKI) facilitates repair and regeneration of damaged tubules.^56^ However, the prolonged activation of Wnt exacerbates fibrotic kidney diseases.^57^ Tubular expression of most of nineteen Wnts and ten frizzled receptors (Fzd) was elevated in fibrotic kidneys of UUO experimental model, except for Wnt5b, Wnt8b, Wnt9b, Fzd4 and Fzd5.^58^ Although conditional deletion of tubular β-catenin exerted no protective effects against tubulointerstitial fibrosis of UUO model,^59^ several antifibrotic treatments interfering with Wnt signaling by diverse strategies were promising. These acknowledged Wnt inhibitors included kallistatin,^60^ dapper3,^61^ dickkopf1 (Dkk1),^58,62^ Dkk2,^63^ ICG-001,^64^ paricalcitol,^65^ secreted frizzled-related protein (Sfrp) 1^66^ and Sfrp4.^67^ As key targets of Wnt ligands, the interstitial fibroblast and pericyte are also recognized in abundant studies. By paracrine pathway, tubule-derived Wnts contribute to the activation of myofibroblasts.^68–70^ What’s more, fibroblast and pericyte canonical Wnt4/β-catenin constitutive activation displayed spontaneous interstitial myofibroblast differentiation even without stimulant or injury.^71^

##### Hedgehog

Hedgehog (Hh) is also a vital mammalian developmental signal pathway that controls tissue/organ patterning, cell growth, and differentiation.^72^ Desert hedgehog (Dhh), Indian hedgehog (Ihh), and Sonic hedgehog (Shh) are three well-known Hh ligands. In human kidney tissues, the ligands of Hh are expressed and secreted in tubule cells, and these interstitial cells with respondency to Hh ligands.^73,74^ And Gli1 is one of Hh target genes.^75^ The lineage-tracing investigation indicated that the expression of Ihh and Shh were increased in UUO surgery-injured tubules.^73,75,76^ As mentioned before, Gli1^+^ MSC-like cells play a key contribution to the myofibroblast pool.^23^ Therefore, Hh signaling participates in the occurrence and progression of kidney fibrosis, mainly via the secretion/release of these ligands from tubular cells and the increase of Gli1 in myofibroblast.

##### SRY-related high-mobility-group box 9 (SOX9)

SOX9 as a transcription factor is responsible for cell growth and differentiation in multiple organs including kidneys.^77^ Kang et al. have found that SOX9-positive cells of kidneys showed progenitor-like functionalities, and contributed to the regeneration of epitheliums after injury.^78^ Some studies have consistently explored that the loss of SOX9 function underlined a failure of survived PT cells to repair after AKI.^79–81^ However, SOX9 is also crucial for kidney fibrosis. Neuron navigator 3 (NAV3)^82^ and homodimer of inhibin subunit beta B (INHBB),^83^ the acting downstream of SOX9, were thought to be potential antifibrotic targets for pharmacological intervention.

##### Estrogen-related receptor alpha (ERRα)

In addition to classical pathways that influence cell differentiation, nuclear receptors such as ERRα were also investigated to maintain the metabolism and differentiation of PT cells via directly control of these cell-specific genes.^84^ Dhillon et al. proved that fatty acid oxidation (FAO) and oxidative phosphorylation in proximal tubules exhibited a positive and reproducible relationship with cell differentiation and disease progression. Furthermore, it was ERRα and peroxisomal proliferation-activated receptor (PPAR) alpha that coupled with metabolism and proximal tubule cell-specific gene expression in mouse and human specimens, while defending against kidney disease of an experimental mouse model.^84^

#### Tubular cell cycle arrest and senescence

Cell cycle arrest is usually beneficial to DNA repair before initiating their proliferation. Both rapid progression through cell cycle phases with incorrect checkpoints, and the prolonged cell cycle arrest, are detrimental for tubules.^85^ Cellular senescence is a fate where cell cycle arrest is permanent and irreversible.^86^ The p16^Ink4a^ and p53-p21^Cip1/Waf1^ are both critical cyclin-dependent kinase (CDK) inhibitors, leading to cellular senescence.^87,88^ Senescent cell is resistant to apoptosis and continually produces a complex secretome as senescence-associated secretory phenotype (SASP) such as proinflammatory and profibrotic mediators, etc.^86^ Numerous studies have cleared that the G1/S and G2/M arrest of tubular cells is crucial drivers in maladaptive repair and kidney fibrosis.^89–91^ Importantly, these senescent cells could be pharmacologically targeted to reduce fibrosis. Recently, several creative studies validated the efficacy and safety of senescent cell clearances, applying transgenic mice with selectively sensitive pharmacological agents,^92,93^ or the mice access to antiapoptotic drugs.^92,94,95^ Moreover, various strategies are being tested to prevent the formation of senescent cells (e.g., exercise, weight loss),^96–98^ trigger the apoptosis of senescent cell (e.g., ABT-263, FOXO4-DRI),^92,99^ reduce the secretion of SASP (e.g., metformin, sirolimus),^100,101^ and take advantage of senescent cell metabolic activity to activate compounds (e.g., galactooligosaccharide conjugated drugs).^102^ In addition, the treatment of CDK4/6 inhibitor PD-0332991, could result in a transient cell cycle arrest after injury, suppressed DNA damage, tubular epithelial cell apoptosis, interstitial inflammatory response, and restored kidney function.^103,104^ The induction of transient cycle arrest or pharmacological quiescence by CDK4/6 inhibition in proximal tubule cells could allow correcting DNA damage with decreasing early apoptosis, senescence as well as AKI-to-CKD transition.

### Immune cells

Immune cells also are the core participants in the fibrotic niche. In tissue specimens from CKD patients, fibrotic niches were associated with infiltrating CD68^+^, myeloperoxidase^+^ (MPO^+^), and CD3^+^ cells. And predominantly CD3^+^ immune cell activity was observed in neighborhoods of adaptive/maladaptive PT and TAL. The positive relationship between specific renal structures and leukocytes was also identified, including MPO^+^ cells with glomeruli and CD68^+^ cells with proximal tubule epithelium.^44^ In mice, normal kidney was governed by tissue-resident macrophages, followed by T and B cells, as well as a relatively small percentage of monocytes, neutrophils, natural killer (NK) cells and dendritic cells (DCs). The myeloid compartment consistently predominated after injury. Whereas, early after ischemia reperfusion injury (IRI) (day 7), the complete knockout of B cells and partial knockout of T cells were observed. If fibrosis occurs after injury (day 28), T cells re-expand.^105^

#### Macrophages

Similar to the tubular epithelium, although monocyte exerts a limited contribution to myofibroblasts,^105^ monocytes/macrophages play critical roles in kidney fibrosis. As acknowledged, the proinflammatory M1 and antiinflammatory M2 macrophage are closely related to organ/tissue injury, repair and fibrosis. Recently, relevant knowledge have been summarized and is not covered in this review.^106^ A new atlas of single-cell RNA transcriptomics has reported that myeloid heterogeneity was involved in the progression and regression of kidney disease. Monocytes recruited to the injured kidney in early stage and adopt rapidly a proinflammatory and profibrotic phenotype that expressed Arginase 1, before transforming to C-C chemokine receptor 21^+^ (Ccr21^+^) macrophages which accumulated in the late stage of kidney damage. However, mannose receptor 1^+^ (Mrc1^+^) and matrix metallopeptidase 12^+^ (Mmp12^+^) macrophages could be characterized by scavenger receptor expression and might contribute to the degradation of matrix.^107^

#### Lymphocytes

A single-cell RNA sequencing of CD4^+^ T cells in fibrotic kidneys uncovered the expansion of T helper 17 (Th17) cells and regulatory T cells. However, the expansion of tissue-resident IL-33R and IL-2Rα positive Tregs before injury could protect kidney damage and scaring.^108^ Tertiary lymphoid tissue (TLT) is inducible ectopic lymphoid tissue in the process of chronic inflammation, which functioned as the priming site of local immune responses.^109^ In the aging kidney, T and B cells, together with resident fibroblasts, form TLTs that cause uncontrollable inflammation and delay tissue repair.^110^ Several studies have documented that TLT is related with the progression of kidney damage.^110–112^ Targeting IL-17A^113^ or CD153/CD30^114^ might slow the progression of kidney diseases by attenuating TLT formation. The natural killer cell is a population of lymphoid cells that exerts crucial functions in innate immunity. Law et al. found an increased number of interstitial CD56^bright^ NK cells (NKp46^+^CD117^+^) in fibrotic kidneys, contributing to tissue scaring by producing interferon (IFN)-γ.^115^

#### Neutrophils

Neutrophils are an important component of innate immune cells. Recently, the most prevalent population of immune cells in the advanced fibrotic kidney is neutrophils by flow cytometric analysis of UUO mice.^116^ TGF-β1 and GM-CSF could trigger neutrophils converting into Siglec-F^+^ (usually seen as an eosinophil marker) neutrophils, which produced profibrotic mediators and secreted collagen-1. Depletion of Siglec-F^+^ neutrophils reduced collagen deposition and disease progression, indicating its promising therapeutic strategy for fibrotic kidney disease.^116^

#### Other immune cells

Dendritic cells and mast cells also participated in kidney fibrosis, and the clearance of DCs or mast cells could alleviate fibrosis.^117,118^ The latest study identified that CXCR2^+^ basophils were recruited by CXCL1 (secreted from profibrotic tubules) as key contributors to kidney fibrosis and suggested that targeting these cells may be a promising guideline for the treatment of CKD.^119^

### ECM network

The ECM network, also termed the decellularized kidney tissue scaffolds, plays an indispensable role in fibrotic niche formation. Using proteomics by unbiased mass spectrometry, researchers decoded almost one thousand ECM proteins, which were divided into four main categories: structural ECM protein (collagens, fibronectin, and elastin), matricellular protein (fibrillin 1 (FBN1), tenascin-C (TNC), connective tissue growth factor (CTGF), and periostin (POSTN)), matrix-modifying proteins and proteoglycans.^16^ Among them, the most increased proteins in fibrotic kidney tissue scaffolds were matricellular proteins, highlighting that they are pivotal contributors in fibrotic niche. TNC, an extracellular matrix glycoprotein, has been confirmed to be a major component of fibrotic niche in renal fibrosis.^120,121^ In adult kidney and other organs, no or little TNC is detected, while the *de novo* expression of TNC is prominent in fibrotic kidneys. TNC, produced primarily by fibroblasts, promotes the activation and proliferation of fibroblasts, as well as ECM production and deposition via activating integrin/adhesive plaque kinase (FAK)/mitogen-activated protein kinase (MAPK) signaling. The knockdown of TNC in vivo inhibited fibroblast activation and proliferation, which in turn reduced renal fibrosis. Further studies revealed that TNC not only activated signaling pathways in fibroblasts, but its unique hexametric structure also acted as a sponge to trap and concentrated other profibrotic factors such as Wnts, thus forming a local microenvironment enriched with the high concentrations of profibrotic mediators, and initiating and accelerating the progression of fibrosis.^120^

## Mechanisms of glomerulosclerosis

Both progressive primary glomerular diseases and secondary glomerular diseases culminate in glomerulosclerosis. In a single-cell transcriptome atlas of glomerulus in mice, five known cell types including podocytes, endothelium, tubules, mesangium, and immune cells are present in glomeruli.^122^ The atlas of human kidneys identifies glomerular endothelial cells, podocytes, mesangial cells, and parietal epithelial cells in glomerulus.^11^ However, some methodological challenges of single-cell technology still exist in glomerular diseases, especially in humans.^123^ For example, the biopsy by core needle provided a small-sized sample, and some specimens are defective to identify glomerular cells.^123^ Moreover, there is a lack of animal experimental models that could well simulate glomerular diseases. Thus, our understanding of the trajectory of glomerulosclerosis is not as clear as that of tubulointerstitial fibrosis.

### Diabetic kidney disease

Chung et al. compared glomerular cells from the leptin-deficient ob/ob obese and lean mice by principal component analysis. The alterations of gene expression were found in mesangial cells and podocytes, but not endothelial cells. The pathway enrichment analysis exhibited the pathway of glucose and lipid metabolism-related gene alterations in both mesangial cells and podocytes. The pathway of apoptotic genes was enriched in podocytes, and the pathway of cell proliferation were triggered in mesangial cells. Furthermore, the number of podocytes decreased whereas the number of mesangial cells increased at 21th week. And it was not surprising to find that the expression of matrix/matrix-modifying proteins was increased in both mesangial cells and podocytes.^124^ However, in another single-cell transcriptomics profiling of DKD experimental model, fewer mesangial cells and podocytes were found in the kidneys of diabetic mice compared to those of control mice, with a greater number of immune cells and glomerular endothelial cells in kidneys of diabetic mice. The data of pathway enrichment analysis from diabetic kidney tissues indicated that the regulation of migration and angiogenesis pathway-related genes was changed in endothelial cells, and the genes involved in the pathway of translation, gene expression, and protein stabilization were highly enriched in mesangial cells. However, a small number of podocytes was captured in the kidneys of diabetic mice. Ctgf, Vegf, Tnfa, and leucine-rich α-2-glycoprotein 1 (LRG1) in diabetic kidneys were highly expressed in glomerular mesangial cells, podocytes, endothelial cells, and immune cells, respectively. The study also found that M1 phenotype macrophages were predominant in glomerular immune cells. Some well-established pairs of ligand-receptor in the crosstalk of glomerular cells were identified such as endothelial Flt1-podocyte Vegfa.^122^ The recent study of early-stage diabetic nephropathy patients by single-nucleus RNA transcriptomics showed no significant difference (*P* = 0.66) in the number of podocytes because of the limited sample number.^125^ The expression podocyte PLA2R1 and THSD7A in early-stage diabetic patients were increased in comparison to a previous study of later-stage diabetes whose PLA2R1 was greatly reduced. For mesangial cells, the angiogenesis-related genes by gene ontology (GO) analysis were enriched which were driven by the increased expression of regulatory genes (MYH9, NR4A1, SLIT3, ADAMTS12) and ECM components (COL4A1, COL4A2). The cluster of cells characterized by VCAM-1, CFH, AKAP12, CLDN1 gene are most likely parietal epithelial cells, whose CFH gene expression being decreased. In endothelial cells, the related genes of ECM components (COL4A1), angiogenesis regulators (VCAM, VEGFC, ITGB1, HDAC9, MYH9, TMEM204, PRCP, MEF2C, NR4A1), and glucose transporters (SLC2A14, SLC2A3) were differentially expressed.^125^ The pairings of ligand-receptor were also investigated among various cell types in glomeruli. In diabetic mesangial cells, CCN1 and SLIT3 expression was elevated. The growth factor-inducible CCN1 controls tissue and organ repair through the interactions with endothelial ITGB3 and podocyte ITGAV, ITGB3, ITGB5.^126^ SLIT3 interacting with podocyte/endothelial cell-expressed ROBO2 regulates cellular migration.^127^ Diabetic podocytes displayed decreased INSR expression, and the NAMPT expression from mesangial cells regulated pancreatic-β cell insulin secretion.^128^ The increased LTBP1 level of diabetic endothelial cells could control the targeting of latent complexes of TGF-β.^129^

### Lupus nephritis

As a secondary glomerular disease, lupus nephritis is a severe manifestation of systemic lupus erythematosus (SLE) associated with a complicated immune mechanism. Single-cell techniques offer new perspectives for understanding this disease. For example, the results from scRNA transcriptomics of human skin and renal biopsies indicated that the analysis of skin as biomarkers could predict kidney disease, because IFN-inducible genes, correlated to chronicity index, immunoglobulin (Ig) G deposition, quantity of proteinuria, and therapeutic response upregulated parallelly in keratinocytes and tubular cells in lupus nephritis.^130^ Furthermore, the high expression of type I interferon-induced genes of tubules could separate proliferative lupus nephritis from membranous symptom.^131^ Another study focused on immune cells revealed that various leukocytes were activated, and explicit interferon responses were detected in most of the cells broadly expressing CX3CR1 and CXCR4. The study also indicated that urine specimens may be a replacement for renal biopsy, in which immune cell gene expression was highly correlated between kidney and urine. Among immune cells, an intrarenal subset of “M2-like” CD16^+^ macrophages with their high CD163 and CXCL12 level was speculated to decoy other immune cells.^132^ Importantly, soluble urine CD163 could be used to distinguish patients with proliferative or membranous lupus nephritis, and also discriminate active lupus nephritis from other forms of SLE patients.^133^ In a model of TLR7-induced mouse lupus, IFN-λ was found to promote systemic immune dysregulation via local consequences in kidney and skin.^134^ In the meanwhile, IFN-λ aggravated lupus-related renal pathological damage and also activated mouse mesangial cells to highly express IFN-stimulated and chemokine genes.^134^

### IgA nephropathy

Multiple ligand-receptor connections were also detected in glomerular cells of IgA nephropathy (IgAN) mice. Glomerular endothelial cells occupied the dominant position by activating and recruiting leukocytes at the initial phase of IgAN.^135^ Among primary glomerular cell types, mesangial cell-expressed Slit3 could activate Robo2 and 4 in podocytes and endothelial cells, respectively. Mesangial cell-expressed secreted phosphoprotein 1 (Spp1) could bind to endothelial sphingosine-1-phosphate receptor 1 (S1pr1). Podocyte-expressed Col1a1 could be binding to endothelial and mesangial cell CD36. At the early stage of IgAN, proximal tubules were surprisingly influenced by the identification of glomerulo-tubular crosstalk.^135^ However, another human study of IgAN emphasized a central role for mesangial cells, which may recognize and transport IgA via the upregulation of joining chain of multimeric IgM and IgA expression.^136^ In addition, mesangial-tubule and mesangial-immune crosstalk also triggered tubulointerstitial inflammation and fibrosis.

### Podocytopathy

Podocyte injury is responsible for proteinuria. Chung et al. identified and validated that Hippo pathway was indispensable to repair podocyte injury. Knockout of the Hippo downstream effector YAP or TAZ led to more prolonged and severe proteinuria and worse glomerulosclerosis.^124^ Focal segmental glomerulosclerosis (FSGS) is initiated from primary, secondary, or genetic podocyte injury. Besides podocytes, parietal epithelial cells were paid more attention in the progression of FSGS. A recent study also found that the transcriptional level of glomerular alpha-2-macroglobulin (A2M) was related to lower proteinuria remission rates, connecting long-term outcomes and endothelial function in FSGS.^137^ Single-cell RNA transcriptomics from urine samples of FSGS patients was also used to investigate disease-related molecular signatures. Renal epithelial and immune cells (predominantly monocytes) were identified in the urine. And shedding of podocytes showed a high level of EMT genes in the urine. Consistent with these results, the genes related to immune signature and EMT signature in urine cells were more highly expressed in kidney biopsies of FSGS patients in comparison to those of minimal change nephropathy (MCD).^138^

## Pathogenesis of vascular rarefaction

Vascular rarefaction, which refers to a decrease in capillary density leading to ischemic and hypoxic conditions, is a pathological feature, a progressive factor, and a consequence of kidney fibrosis. Vascular rarefaction is related to the apoptosis, detachment, and dysfunction of endothelial cells.^139^ Previous studies mainly concentrated on the dysfunction of angiogenic mediators such as VEGF, which is an important factor of vascular repair and angiogenesis. The level of VEGF was decreased in chronic ischemic kidney disease, resulting in kidney function deterioration and microvascular rarefaction.^140^ However, VEGF is also a key anti-tumor drug target and the restoration of VEGF might pose a risk.^141^ Therefore, other mechanisms regulating vascular rarefaction need to be discovered.

As the other family member of angiogenic growth factor, angiopoietins (Angpt) control vascular stability and maturation.^142^ Inflammation and hypoxia triggered endothelial cell angpt2 expression,^143^ which could destabilized the integrity of endothelium.^144^ A latest published study has proven that angpt2 inhibition by angpt1 or a Fc-fusion peptide L1-10 could attenuate endothelial inflammation, apoptosis, and renal fibrosis in progressive kidney diseases.^145^ The LRG1 as an angiogenic factor could promotes angiogenesis of myocardial infarction and ocular disease mouse model.^146,147^ Liu et al. reported that plasma and kidney LRG1 expression was increased in CKD patients and proved that LRG1 regulated capillary-like formation. Gene knockout of LRG1 exacerbated capillary rarefaction, inflammatory cells infiltration, and kidney fibrosis following UUO surgery.^148^

Caspase-3 is a well-known effector of programmed cell death or cell apoptosis.^149^ Active caspase-3 has capacity to regulate apoptosis during kidney injury.^150^ It is interesting to find that, in caspase-3 knockout mice, tubular damage and kidney dysfunction are worsening in early-stage IRI, while microvascular integrity is alleviated during overall process of disease. The caspase-3 gene knockout mice exhibit the remission of tubular damage, microvascular dropout, and tubulointerstitial fibrosis in the long term. These findings confirm the significance of caspase-3 in controlling IRI-induced kidney fibrosis and microvascular endothelial cell apoptosis.^151^ Activin A receptor like type 1 (ALK1) as a type I receptor for TGF-β family proteins regulated the ECM accumulation and organ fibrosis in the liver, heart, skin, and kidneys.^152,153^ In a recent study of ALK1 heterozygous mice, the recovery of fibrotic kidney benefited by myofibroblast clearance and low degree of vascular rarefaction, indicating that ALK1 deficiency ameliorated UUO-injured peritubular microvasculature.^152^

Klotho is a well-known renoprotective gene/protein. Recently, a study reveals a promising mechanism by which klotho improved peritubular capillary rarefaction and delayed renal fibrosis. The study concluded that VEGFR2 was indispensable for vascular integrity, where Klotho exerted antifibrotic and vascular protective effects partially through the modulation of VEGFR2 function in kidneys.^154^

As above-mentioned, FBN1 as a component of ECM network, is increased in the kidneys of CKD animals and patients. Li et al. recently found that FBN1 inhibited endothelial cell proliferation and induced their apoptosis in vitro. Furthermore, FBN1-triggered endothelial injury could be abolished by the suppression of integrin αvβ6/TGF-β pathway and FBN1 blockage mitigated vascular rarefaction and ameliorated fibrotic scaring in kidneys.^155^ The results identify FBN1 as a mediator to orchestrate a detrimental microenvironment for endothelial cells in vascular rarefaction. Lysyl oxidase (LOX) catalyzes the crosslinking of ECM proteins and plays a key function in stabilizing resistant matrix degradation.^156^ Not unexpectedly, LOX takes part in the process of renal fibrosis. Recently, a study observed that LOX inhibition could partially restore microvascular rarefaction, inhibit the loss of pericyte and maintain endothelial cell-pericyte interaction in UUO-induced fibrotic kidneys.^157^

Pericytes embedded in capillary basement membrane could directly communicate with endothelial cells. Pericytes contribute to microvascular stability and modulate cortical/medullary flow by dilating/contracting in response to stimuli, which were produced by neighboring tubular and endothelial cells.^158^ A study showed that complement activation in IRI caused the occurrence of pericyte-to-myofibroblast and the decreased area of peritubular capillaries lumen. Of note, complement 5a exerted profibrotic capacity to drive pericytes toward maladaptive phenotype via the activation of ERK.^159^ Another study explored that bone marrow-derived mesenchymal stem cells (BMSCs) transplantation could improve pericyte vitality and suppress pericyte detachment and trans-differentiation. The direct BMSCs differentiation into pericytes by the transplantation of BMSCs also takes part in AKI-induced peritubular capillary repair, archiving the effect of preserving renal function and reducing kidney fibrosis.^160^

It is exciting to find that a sodium-glucose cotransporter 2 (SGLT2) inhibitor Luseogliflozin ameliorate renal interstitial fibrosis and peritubular capillary rarefaction in animal models.^161^ And a bioengineered fusion of elastin-like polypeptide with VEGF was also designed to restore kidney function of renovascular disease and increase renal microvascular density.^162,163^ However, there are data from single-cell technology about vascular rarefaction, so advances in technology will be a tremendous help in understanding the mechanisms that vascular rarefaction occurs.

## Epigenetics in kidney fibrosis

Conrad Waddington for the first time reported ‘epigenetics’ to illuminate genes interacting with the environment. Epigenetics is increasingly explored as one of the major determinants in the kinds of miscellaneous diseases.^13^ Epigenetics is a heritable alteration in the expression pattern of genes, which is caused by a mechanism other than the changes in DNA sequence. The RNA interference, chromatin remodeling, and DNA methylation are all well-known epigenetic mechanisms.^14^ As for the patients of CKD, the alteration of many factors including oxidative stress, inflammation, uremic toxins, and metabolic state such as hyperglycemia, could control genome epigenetic reprogramming, further contributing to the progression of kidney disease.^164^

### Metabolic and hypoxic memory

DKD is a typical CKD affected by environmental and genetic factors. Considerable studies indicate that the DKD-featured gene expressions are modulated by classical signaling, but also by epigenomics being responded to environmental changes. Notably, the epigenetic mechanism, which is termed as “metabolic memory”, could regulate perpetually a long-term statement of DKD-associated genes and phenotypes initiated priorly by hyperglycemia, regardless of consequent blood glucose control.^164^ Therefore, controlling the occurrence of epigenetic events in early-stage DKD might be worthy of early diagnosis and timely treatment, which is believed to delay or stop disease progression. With the technology of epigenome-wide association studies (EWASs), the epigenetic signatures of DKD patients could be valuable for personalized precision medicines. In addition to epigenetic mechanisms shared with tubulointerstitial fibrosis, unique epigenetics, and epigenomics also play important roles in DKD.^165^

Like metabolic memory, “hypoxic memory” has been proposed to understand the AKI-to-CKD transition.^166^ During and after AKI episodes, hypoxia sustainedly exists owing to capillary rarefaction. Hypoxia triggers sterile inflammatory response and fibrotic scaring, and in turn kidney fibrosis exacerbates the degree of tissue hypoxia. As known, renal fibrosis caused capillary loss and intensified the distance between tubular cells and capillaries, resulting in the decreased efficiency of oxygen diffusion.^166–169^ Moreover, hypoxia-induced epigenetic alterations including chromosome conformational change, histone modification (e.g., demethylation of H3K9me2 and methylation of H3K27me3), DNA methylation, and these changes of non-coding RNAs (ncRNAs).^166^ In kidneys, hypoxia is recorded as epigenetic alterations which are termed as “hypoxic memory”, and exerted long-term effects after the recovery from the initial AKI episode. Therefore, targeting hypoxic memory are a potential strategy to delay or stop the AKI-to-CKD transition.

### DNA methylation

DNA methylation is a reaction by adding a methyl group to carbon 5 position of the cytosine residue (CpG site) which is catalyzed by DNA methyltransferases (DNMTs), usually contributing to the loss of gene expression.^170^ Hypomethylation or DNA methylation on specific CpG site exert key functions in kidney development, whereas abnormal methylation or hypermethylation also could happen in various kidney diseases. Recently, Susztak et al. reported the genotype and DNA methylation profiles of more than 500 kidney samples and identified approximately 140,000 CpG with methylation QTLs (meQTLs).^171^ In this study, methylation variation explained a larger percentage of heritability than gene expression. DNA methylation-mediated heritability was significantly tissue-specific and enriched in kidney-specific enhancer regions.^171^ A study of cytosine methylation in CKD patients discovered these differentially methylated loci in tubules and significant difference of methylation was found in 1061 genes, many of which are known in kidney fibrosis such as TGFβ receptor 3 (TGFBR3), SMAD3, and SMAD6.^172^ Some of the genes, like hepatocyte nuclear factor (HNF)^173^ and SIX2,^174^ are involved in renal transcription factors, and other genes, like collagens and laminins, are indicators of cell adhesion pathways. Moreover, 19 differential CpG sites of whole-blood DNA methylation in an EWAS of two population-based cohorts including 4,859 participants were significantly and reproducibly related to eGFR or CKD, as well as kidney fibrosis clinical endpoint.^175^ In the corresponding kidney biopsies, the gene DNA methylations of protein tyrosine phosphatase non-receptor type 6 (PTPN6)/prohibitin 2 (PHB2), ankyrin repeat domain containing 11 (ANKRD11), trinucleotide repeat containing 18 (TNRC18), solute carrier family 66 member 1 (SLC66A1), and pre-mRNA processing factor 8 (PRPF8) were significantly correlated with the degree of fibrosis.^175^ Another genome-wide study reported that DNA hypomethylation and hypermethylation were present at different loci in whole-blood of CKD patients.^176^ Previously, the differentially methylated genes containing engulfment and cell motility 1 (ELMO1), cut like homeobox 1 (CUX1), FKBP5, protein tyrosine phosphatase receptor type N2 (PTPRN2), and inhibin subunit beta A antisense RNA 1 (INHBA-AS1) have been concluded in transcription regulation, signaling, and apoptosis pathway.^176^ Notably, the consistent trend of the correlation between kidney function and DNA methylation was shown in both whole-blood samples and kidneys, suggesting that the profiles of whole-blood DNA methylation could reflect the DNA methylation in kidneys.

Additionally, the methylation of many specific genes also is closely related with the progression of CKD. At first, ECM-related genes could be modulated by their corresponding promoters’ methylation. For example, SMAD3 and SMAD6 methylated changes were correlated with their transcriptional levels in samples of CKD pateints.^172^ The RAS protein activator-like 1 (RASAL1) hypermethylation was related to kidney fibrosis with the perpetuation of myofibroblast activation, where TGF-β mediated the hypermethylation of RASAL1 with DNMT1 effect in fibrogenesis.^177^ In whole-kidney biopsy and fibroblasts, hypermethylation and transcriptional silencing of RASAL1 was also related to severe fibrosis. DNA methylation could be inherited by progeny, which could explain the perpetuation of myofibroblast activation.^177^ Moreover, hypermethylation of antifibrotic genes like secreted frizzled-related proteins 5 (sFRP5),^178^ KLOTHO,^179,180^ and Krüppel-like factor 4 (KLF4) also contribute to renal fibrosis.^181^ Collectively, targeting DNA methylation might be a therapeutic strategy, as RASAL1 hypermethylation could be revoked by bone morphogenic protein 7 (BMP-7), which is a well-known endogenous antagonist of TGF-β.^182^

### Histone acetylation

The acetylation and methylation of lysine residues are the most important two kinds of histone modifications.^164^ In most cases, histone acetylation mediated by histone acetyltransferases (HATs) could promote transcriptional activation, while histone deacetylases (HDACs)-mediated histone deacetylation could repress gene expression.^183^ Histone methylation with the different modified lysine residues could function as gene transcription activation or repression, respectively.^184^ Beyond those, histone phosphorylation, ubiquitylation, and glycosylation are extensively investigated, and their modified location also spreads to serine, threonine, and arginine residues.^185^

Histone acetylation could facilitate chromatin opening, which DNA is more accessible to the transcription machinery and promote gene transcription.^186^ Histone acetylation could be reversibly modulated by HATs, HDACs, and bromodomain and extraterminal (BET) proteins (the “readers” of lysine acetylation),^187^ which participate in various pathophysiological events of kidney fibrosis such as partial EMT,^188^ myofibroblast activation,^189,190^ inflammation,^191,192^ and profibrotic factor secration.^193,194^ The HATs mainly included the P300/CREB binding protein (CBP) and P300/CBP-associated factor (PCAF). P300 inhibitors, L002,^195^ C646,^196,197^ and C66^198^ reduced renal fibrosis in the hypertensive and diabetic kidneys. Garcinol, a PACF inhibitor, alleviated kidney fibrosis of UUO model by inhibiting the activation of NF-κB and NRF2.^193^

#### Histone deacetylases

There are four categories of HDACs containing 18 members. The HDACs of class I includes four subtypes of 1, 2, 3, and 8; The class II HDACs contain two subclasses IIa (4, 5, 7, and 9) and class IIb (6, 10). Sirtuin (SIRT) 1–7 are the members of III HDACs, and HDAC11 is only one of IV HDAC.^187^ The pan HDAC I and II inhibitor Trichostatin A could delay fibrotic kidney via suppressing TGF-β1 and JNK/Notch2 pathways,^190,199^ as well as preserving E-cadherin expression.^200^ The selective HDAC I inhibitor MS-275 retarded fibrosis by suppressing Smad3 and STAT3 phosphorylation, and reduced TGF-β1 expression in UUO-injured kidneys.^201^ However, some natural and synthetic compounds activating SIRTs also present renoprotective effects, where resveratrol and honokiol activate SIRT1 and SIRT3, respectively. Activating SIRT1 by resveratrol reduces interstitial fibrosis via suppressing acetylated Smad3 and MMP-7 in UUO mice.^202,203^ Honokiol alleviated the acetylation level and kidney fibrosis, whose mechanism was related to the SIRT3-mediated metabolic reprogramming.^204,205^ In addition, honokiol attenuated angiotensin II (Ang II)-triggered kidney dysfunction and fibrotic scaring by inhibiting KLF15-mediated ECM protein expression.^206^ SRT1720, SRT2183, and SRT3025 are all synthetic SIRT1 activators, showing antifibrotic effects in CKD models.^207–209^ SIRT6 was also found to improve renal fibrosis by epigenetically suppressing β-catenin signaling.^210^ These results are consistent with those obtained in SIRT1, SIRT3, or SIRT6 genetically engineered mice.^210–212^ However, SIRT2 seems to play the other way around. He et al. reported that chemical inhibition and genetic knockdown of SIRT2 alleviated TGF-β1-triggered activation of fibroblast and tubulointerstitial fibrosis via suppressing murine double minute 2 (MDM2) expression.^213^

Up to now, several isoform-selective HDAC inhibitors have been developed, which are beneficial to individual HDAC member functional studies in fibrotic kidneys. RGFP966 as an HDAC3-selective inhibitor could improve kidney fibrotic damage in both aristolochic acid nephropathy and UUO mice.^214^ PCI34051, a highly selective inhibitor of HDAC8, decreased the number of tubular epithelial cell G2/M-phase arrest as well as suppressed UUO surgery-induced Smad3, STAT3, β-catenin phosphorylation and Snail expression.^188,200^ Furthermore, inhibition of HDAC8 restored the reduction of Klotho and BMP-7 in UUO experimental model. HDAC4 inhibition by MC1568 also decreased the level of profibrotic factors and preserved Klotho, BMP-7, and Smad7 expression.^188^ Tubastatin A and ACY-1215 as HDAC6 inhibitors presented antifibrotic effects in different CKD models.^215,216^ Quisinostat alleviated renal fibrosis in UUO and Ang II-induced hypertension by inhibiting HDAC11 and subsequently repressing KLF15.^217^

#### Bromodomain and extra terminal proteins

BET proteins as epigenetic readers contain Brd2-4, and Brdt.^218^ Increasing studies report that BET proteins could modulate kinds of cell functions such as pericyte/fibroblast activation, inflammation, cell growth, and differentiation.^218,219^ Using JQ1, I-BET151, and ZLD2218 to inhibit BET protein could prevent the inflammatory response, inhibit G2/M-phase cell arrest, and suppress profibrotic signaling activation, contributing to the delay of renal fibrosis.^220–223^

#### Acetylation of non-histone proteins

Unlike demethylated agents against progressive CKD, the HATs, HDACs, and BET inhibitors have all demonstrated therapeutic effects on renal fibrosis, which indicates other mechanisms beyond histone acetylation exist. For example, STAT3 and NF-κB could be acetylated in mammalian cells.^224^ Non-histone protein acetylation could mediate various functions including enzymatic activity, protein stability, and the interactions of protein-protein or protein-DNA.^225^

### Histone methylation

**Fig. 2:**
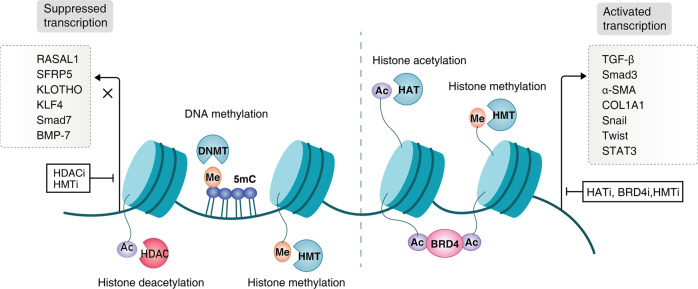
Histone modification and DNA methylation in kidney fibrosis. Suppression of antifibrotic genes (e.g., RASAL1, KLOTHO, KLF4, Smad7) could be accomplished by DNA methyltransferase (DNMT)-governed DNA methylation, HDACs-induced deacetylation, and histone methyltransferase (HMT)-driven methylation (e.g., H3K9me, H3K27me). On the contrary, HATs-mediated histone acetylation, HMT-ruled H3K4me, and BET proteins are involved in profibrotic gene transcriptional activation (TGF-β, Smad3, α-SMA, Snail, Twist, STAT3, et al.) α-SMA alpha-smooth-muscle actin, BMP-7 bone morphogenic protein 7, BRD4 bromodomain-containing protein 4, Col1a collagen-1α, KLF4 Krüppel-like factor 4, RASAL1 RAS protein activator like 1, SFRP5 secreted frizzled-related proteins 5, STAT3 signal transducer and activator of transcription 3, TGF-β transforming growth factor beta

As known, histone lysine (K) or arginine (R) methylation are also essential epigenetic modifications to control gene expression in CKD, which are mainly regulated by methyltransferases. The methyltransferases could mediate substrate-specific methylation at one or two lysine residue(s) in a single histone.^226^ The methylation effects do not alter the charge of histone and not interfere with DNA association, which controls gene transcription via histone readers.^227^ In Fig. 2 and Table 2, the functions of histone methylation and methyltransferase in kidney fibrosis are summarized.

**Table 2:** Histone methylation and methyltransferases in kidney fibrosis

Methyltransferase	Histone site	Interventions	Experimental model	Results	Ref.
DOT1L	H3K79me2	EPZ5676	UUO	TGF-β↓, G2/M arrest↓, Snail↓, Twist↓, Notch↓, SMAD3↓, EGFR↓, PDGFR↓, STAT3↓, AKT↓, NF-κB↓, KLOTHO↑, SMAD7↑	^289^
	H3K79	EPZ004777	IRI	ROS↓, PI3K/AKT↓	^290^
G9a	H3K9me1	BIX01294	UUO	α-SMA↓, fibronectin↓, KLOTHO↑	^291^
SMYD2	H3K36me3	AZ505	UUO	SMAD3↓, ERK1/2↓, AKT↓, STAT3↓, NF-kB↓, Snail↓, Twist↓, SMAD7↑	^292^
SETD7	H3K4	PFI-2	folic acid, UUO	M2 macrophages-myofibroblasts transition↓, myeloid fibroblasts activation↓, inflammation↓	^293^
	H3K4me1	sinefungin	UUO	mesenchymal markers↓, ECM↓	^294^
	H3K4me2	-	type 1 diabetes (STZ)	Col1a↑	^295^
SUV39H1	H3K9me2	-	type 1 diabetes (STZ)	Col1a↑	^295^
EZH2	H3k27me3	3-DZNeP, GSK126	UUO	SMAD7↑, PTEN↑	^296^
	H3k27me3	3-DZNeP	hyperuricemia	serum uric acid↓, XOD↓, TGF-β/Smad3↓, EGFR↓, ERK1/2↓, EMT↓, apoptosis↓, fibroblast activation↓, ECM↓, NF-κB↓	^297^
	H3k27me3	GNA	UUO, folic acid	Smad7↑, TGF-β/Smad3↓, α-SMA↓, fibronectin↓, collagens↓	^298^
PRMT1	H4R3Me2a	AMI-1	UUO	TGF-β/Smad3↓	^299^
MLL1/WDR5	H3K4me3	MM-102, OICR-9429	IRI	p16↓	^300^

*AKT* protein kinase B, *α-SMA* alpha-smooth-muscle actin, *Col1a* collagen-1α, *DOT1L* disruptor of telomeric silencing 1-like, *ECM* extracellular matrix, *EGFR* epidermal growth factor receptor, *EMT* epithelial-to-mesenchymal transition, *ERK* extracellular signal-regulated kinase, *EZH2* enhancer of zester homolog 2, *GNA* gambogenic acid, *IRI* ischemia-reperfusion injury, *MLL1* mixed-lineage leukemia 1, *NF*-*κB* nuclear factor kappa B, *PI3K* phosphatidylinositol 3-kinase, *PDGFR* platelet-derived growth factor receptor, *PRMT*-1 protein arginine methyltransferase 1, *PTEN* phosphatase and tensin homolog, *ROS* reactive oxygen species, *STED7* SET domain containing 7 histone lysine methyltransferase, *SMYD2* SET and MYND domain containing 2, *STAT3* signal transducer and activator of transcription 3, *STZ* streptozotocin, *TGF*-β transforming growth factor beta, *UUO* unilateral ureteral occlusion, *WDR5* WD repeat domain 5, *XOD* xanthine oxidase, 3-*DZNeP* 3-deazaneplanocin A

### Other histone modifications

Histone H2AX serine 139 phosphorylation (γH2AX) is a common histone modification, which is induced by ATM, ATR, and DNA-PKs.^228^ The γH2AX is an important DNA damage marker and identified in DKD and AKI.^229,230^ Importantly, DNA damage is closely associated with proximal tubular maladaptive repair and resultant kidney fibrogenesis. Additionally, the crotonylation and lactylation of histone also take part in kidney diseases, which are gradually gaining attention.^231^

### Non-coding RNAs

The ncRNAs are a kind of functional RNA molecule, and mainly contained miRNAs, lncRNAs, circRNAs, small nucleolar RNAs (snoRNAs), and transfer RNAs (tRNAs).^232^ Accumulating evidence suggests that miRNA and lncRNA in kidneys play important roles in damage, repair, and fibrosis.^233^

#### miRNA

The miRNAs are a class of evolutionarily conserved and small ncRNAs, contributing to the control of translation and mRNA degradation.^234^ The miRNA could theoretically target masses of mRNAs to exert diverse functions such as regulating development, differentiation, apoptosis, and stress.^235^ The role of microRNAs in kidney fibrosis is different: some miRNAs are fibrotic promotors and some are repressors.^236^ Here, we summarize miRNAs in both humans and mice (Table 3).

**Table 3:** Role of microRNAs in kidney fibrosis

microRNA	Targets	Ref.
Pro-fibrosis		
miR-21	TGF-β1/Smad3, Smad7, MMP9/TIMP1, PTEN/Akt, PPARα, Mpv17l, Cdc25a/Cdk6, Spry1	^301–307^
miR-33	CPT1α, CROT, HADHB	^308^
miR-34a	KLOTHO, Bcl-2	^309,310^
miR-132	TGF-β signaling, STAT3/ERK pathways	^311^
miR-150	SOCS1	^312^
miR-192	MDM2/p53, Zeb1, ZEB2/TGF-β/CTGF, GLP-1R	^313–317^
miR-214	ULK1, PTEN, E-cadherin, mt-Nd6, mt-Nd4l	^318–321^
miR-324	Prep	^322^
miR-382	HSPD1, PTEN/Akt, SIRP-α/STAT3, kallikrein 5, SOD-2	^323–327^
miR-433	Azin1, TGF-β/Smad3	^328^
Anti-fibrosis		
miR-29	Col1-4, TPM1, HDAC4, YY1, TGF-β3, PI3K/AKT	^329–333^
miR-30	SOX9, CTGF, Snail1, Snail2, Zeb2	^334–336^
miR-181	Egr1	^337^
miR-200	ZEB1, ZEB2, Snail2, KEAP1, TGF-β1	^338–340^
miR-204	PTEN11/p-STAT3, SP1	^341,342^
Let-7	Col1a2, Col4a1, TNFAIP3	^343,344^

*AKT* protein kinase B, *Azin1* antizyme inhibitor 1, *Cdc25a* cell division cycle 25a, *Cdk6* cyclin-dependent kinase 6, *Col* collagen, *CPT1α* carnitine palmitoyltransferase 1 A, *CROT* carnitine O-octanoyltransferase, *CTGF* connective tissue growth factor, *Egr1* early growth response 1, *ERK* extracellular signal-regulated kinase, *GLP-1R* glucagon like peptide 1 receptor, *HADHB* hydroxyacyl-CoA dehydrogenase trifunctional multienzyme complex subunit beta, *HDAC4* histone deacetylase 4, *HSPD1* heat shock protein family D (Hsp60) member 1, Keap1 kelch like ECH associated protein-1, *MDM2* murine double minute 2, *MMP* matrix metallopeptidase, *Mpv17l* MPV17 mitochondrial inner membrane protein like, *mt-Nd* mitochondrial genes-NADH-dehydrogenase, *PI3K* phosphatidylinositol 3-kinase, *PPARα* peroxisome proliferator activated receptor alpha, *Prep* prolyl endopeptidase, *PTEN* phosphatase and tensin homolog, *SIRP-α* signal regulatory protein alpha, *SOCS1* suppressor of cytokine signaling-1, *SOD-2* superoxide dismutase 2, *SOX9* SRY-box transcription factor 9, *Spry1* sprout RTK signaling antagonist 1, *STAT3* signal transducer and activator of transcription 3, *STZ* streptozotocin, *TGF-β* transforming growth factor beta, *TIMP* tissue inhibitor of matrix metalloproteinase, *TNFAIP3* tumor necrosis factor alpha-induced protein 3, *TPM1* tropomyosin 1, *ULK1* unc-51 like autophagy activating kinase 1, *YY1* Yin Yang 1, *ZEB* zinc finger E-box binding homeobox

#### lncRNA

The lncRNAs are a kind of conserved non-coding transcripts with >200 nucleotides, which are not translated into protein with little or no open reading frame.^237^ In fact, lncRNAs exert multiple biological functions by binding to RNA, DNA, or protein. Cytoplasm lncRNA could function as competing endogenous RNA (ceRNA) and modulate the stability, degradation, and translation of mRNAs to control gene expression. The lncRNAs localized in the nucleus could modulate chromosome conformation, and the rate of gene transcription activation.^237^ Recently, Xia et al. have summarized the effects of lncRNA on renal fibrosis and not covered in detail herein.^238^

## Biomarkers and therapeutic medicines for kidney fibrosis

### Novel biomarkers

**Fig. 3:**
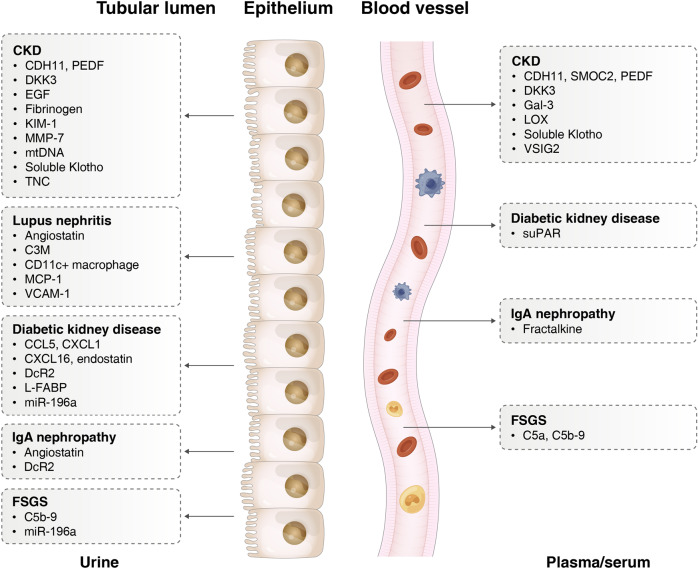
Non-invasive biomarkers of kidney fibrosis in urine and blood. Blood or urinary biomarkers that are associated with biopsy-proven kidney fibrosis. CCL5 C-C motif chemokine ligand 5, CDH11 Cadherin-11, CXCL C-X-C motif chemokine ligand, DcR2 decoy receptor 2, DKK3 Dickkopf-3, EGF epidermal growth factor, Gal-3 Galectin-3, KIM-1 kidney injury molecule 1, L-FABP liver fatty acid binding protein, LOX lysyl oxidase, MCP-1 monocyte chemoattractant protein-1, MMP-7 matrix metalloproteinase 7, mtDNA mitochondrial DNA, PEDF pigment epithelium-derived factor, PTC-EMP peritubular capillary-endothelial microparticles, SMOC2 Sparc-related modular calcium binding protein-2, suPAR soluble urokinase plasminogen activator receptor, TNC Tenascin-C, VCAM-1 vascular cell adhesion molecule 1, VSIG2 V-set and immunoglobulin domain containing 2

As diagnostic criteria for CKD, the GFR, and albuminuria are not good at assessing the degree of renal fibrosis. Although tubulointerstitial fibrosis is well established to correlate with renal dysfunction, fibrotic initiation in kidneys usually happens before the GFR decrease.^239^ And albuminuria mainly reflects glomerular injury. Up to now, the biopsy still is a gold standard for investigating kidney fibrosis with the limitation of invasive detection. Therefore, quantified biomarkers of plasma or urine specimens are very valuable for evaluating the degree of fibrosis. Although many biomarkers have been found to reflect renal function or predict GFR decline, their direct relationship to kidney fibrosis has not been demonstrated. Here, we summarized blood or urinary biomarkers that are associated with biopsy-proved kidney fibrosis (Fig. 3, Table 4).

**Table 4:** The blood or urinary molecules as non-invasive biomarkers of kidney fibrosis

Biomarker	Relationship in kidney fibrosis	Ref.
Angiostatin	The levels of urinary angiostatin/creatinine in patients are associated with tubular atrophy/interstitial fibrosis	^345^
C3M	C3M in urine inversely correlates with tubular atrophy/interstitial fibrosis	^346^
C5a, C5b-9	The plasma C5a level is correlated with the proportion of segmental sclerotic glomeruli. The plasma and urinary levels of C5b-9 are correlated with interstitial fibrosis	^347^
CCL5, CXCL1	Urinary sediment CCL5 and CXCL1 mRNAs are associated with interstitial fibrosis degree	^348^
CD11c^+^ macrophages	The numbers and proportions of urinary CD11c^+^ macrophages are associated with the values of chronicity indices such as tubular atrophy/interstitial fibrosis	^349^
CDH11, SMOC2, PEDF	Higher levels of plasma CDH11, SMOC2, and PEDF and urinary CDH11 and PEDF are significantly associated with increasing severity of interstitial fibrosis and tubular atrophy	^350^
CXCL16, Endostatin	Urinary CXCL16 and endostatin are correlated with tubular atrophy/interstitial fibrosis score	^351^
DcR2	Urinary DcR2 level is correlated with scores of tubulointerstitial injury	^352,353^
DKK3	Urinary DKK3 is related to the extent of tubulointerstitial fibrosis. DKK3 in serum is associated with higher chronicity index at biopsy and flares rate	^354–356^
EGF	Urinary EGF shows significant correlation with intrarenal EGF mRNA, and tubular atrophy/interstitial fibrosis	^357^
L-FABP	Urinary L-FABP is positively associated with interstitial fibrosis	^358^
Fibrinogen	Urinary fibrinogen correlates with tubular atrophy/interstitial fibrosis	^359^
Fractalkine	Plasma fractalkine is associated with mesangial hypercellularity pathological damage, CD68^+^ macrophage, and CD20^+^ B cell infiltration in renal tissue and renal outcome in IgAN patients	^360^
Gal-3	Higher plasma Gal-3 levels are associated with interstitial fibrosis, tubular atrophy, and vascular intimal fibrosis	^361^
KIM-1	Urinary KIM-1 is associated with inflammation, renal function, and also reflects KIM-1 in tissue. Plasma KIM-1 is also associated with tubular atrophy/interstitial fibrosis	^362,363^
LOX	Serum LOX levels correlates with the area of kidney fibrosis	^364^
MMP-7	Urinary MMP-7 correlates with renal fibrosis scores in CKD patients	^365^
miR-196a	Urinary miR-196a is associated with glomerular sclerosis and tubular atrophy/interstitial fibrosis	^366,367^
MCP-1	Urinary MCP-1 correlates with chronic damage, especially fibrous crescents, and tubular atrophy/interstitial fibrosis	^368^
mtDNA	Urinary mtDNA has positive correlations with interstitial fibrosis	^369,370^
PTC-EMPs	Urinary PTC-EMPs levels correlates directly with interstitial fibrosis, and inversely with the number of peritubular capillary	^371^
Soluble klotho	High serum klotho is associated with decreased odds ratios of interstitial fibrosis and segmental sclerosis. Patients with a lower urinary klotho-to-creatinine ratio are more likely to have diffuse foot process effacement	^372,373^
suPAR	Serum suPAR is correlated with tubular atrophy/interstitial fibrosis score and interstitial inflammation score	^374^
TNC	There is a robust correlation between urinary TNC and kidney fibrotic score	^121^
VCAM-1	Urinary VCAM-1 is associated with fibrous crescents	^375,376^
VSIG2	Plasma VSIG2 is positively associated with interstitial fibrosis/tubular atrophy	^362^

*CCL5* C-C motif chemokine ligand 5, *CDH11* Cadherin-11, *CKD* chronic kidney disease, *CXCL* C-X-C motif chemokine ligand, *DcR2* decoy receptor 2, *DKK3* Dickkopf-3, *EGF* epidermal growth factor, *Gal-3* Galectin-3, *IgAN* IgA nephropathy, *KIM-1* kidney injury molecule 1, *L-FABP* liver fatty acid binding protein, *LOX* lysyl oxidase, *MCP-1* monocyte chemoattractant protein-1, *MMP-7* matrix metalloproteinase 7, *mtDNA* mitochondrial DNA, *PEDF* pigment epithelium-derived factor, *PTC-EMP* peritubular capillary-endothelial microparticles, *SMOC2* Sparc-related modular calcium binding protein-2, SUPER soluble urokinase plasminogen activator receptor, *TNC* Tenascin-C, *VCAM-1* vascular cell adhesion molecule 1, *VSIG2* V-set and immunoglobulin domain containing 2

### Treatment of kidney fibrosis or CKD

With the help of gene editing and high-throughput sequencing technology, more kidney fibrotic mechanisms are continually explored. Generally, the potential of the therapeutic drugs will come from the target of these crucial mechanisms. Unfortunately, no effective drugs at present exist against kidney fibrosis. The renin-angiotensin system (RAS) blockade,^8,240,241^ SGLT2 inhibitor,^242,243^ GLP-1 receptor agonist,^244,245^ Atrasentan (endothelin-1 blocker),^246^ Tolvaptan (vasopressin receptor 2 antagonist)^247^ and Finerenone (non-steroidal anti-mineralocorticoid)^248^ can delay the progression of CKD (especially in DKD) to varying degrees. In fact, they are not designed to treat CKD. Some studies have revealed their antiinflammatory, and antifibrotic effects in cultured cells and animal models.^249–251^ Herein, we summarized therapeutic strategies tested in clinical trials targeting fibrotic drivers and signal pathways of tubular damage, regeneration, and inflammatory response (Table 5). It is also important to note that targeting single pathogenetic phenotype might lead to unpredictable events in the co-occurring process.

**Table 5:** Clinical trials of antifibrotic drug in CKD

Drug	Target	Disease	Phase	Outcome	ClinicalTrials.gov Identifier
FG-3019	CTGF	FSGS	1	Terminated	NCT00782561
		Microalbuminuric DKD	1	Treatment was well tolerated; Decrease in albuminuria	NCT00102297
		DKD with urine ACR > 200 mg/g	1	Unknown	NCT00754143
		Type 2 diabetes and DKD with urine ACR > 200–3000 mg/g	2	Terminated early for business purposes	NCT00913393
RG-012	miR-21	Alport syndrome	1	Unknown	NCT03373786
Bardoxolone methyl	Nrf2	CKD at risk of rapid progression	2	Increase in the eGFR	NCT04702997
		Patients with advanced CKD and type 2 diabetes	2	Increase in the eGFR	NCT00811889 (BEAM study)
		CKD patients with type 2 diabetes	2	Increase in measured GFR	NCT02316821 (TSUBAKI study)
		IgAN, CKD associated with type 1 diabetes, FSGS, ADPKD	2	Increase in the eGFR	NCT03366337
		Patients with type 2 diabetes and stage 4 CKD	3	No reduction in the risk of ESKD or death from cardiovascular causes; Increased rate of cardiovascular events	NCT01351675 (BEACON study)
		Alport syndrome	2/3	Preservation in eGFR	NCT03019185 (CARDINAL study)
Pirfenidone	TGF-β	FSGS	2	Decreased GFR loss; No change in proteinuria	NCT00001959
		DKD	1/2	GFR increased in the pirfenidone 1200-mg/d group	NCT00063583
		DKD	3	Unknown	NCT02689778
GCS-100	Galectin-3	CKD	1	Unknown	NCT01717248
		CKD	2	Unknown	NCT01843790, NCT02155673
		DKD	2	Unknown	NCT02312050
Apabetalone	BET	Fabry Disease	1/2	Unknown	NCT03228940
		CKD subjects with a history of CVD	2	Apabetalone was well tolerated; Favorable effects of apabetalone on ALP and eGFR	Post-hoc analysis of NCT01423188 (SUSTAIN study) and NCT01067820 (ASSURE study)
		Patients with CKD and type 2 diabetes	3	Apabetalone was associated with a reduced hazard for MACE and heart failure hospitalization	CKD subgroup analysis of NCT02586155 (BETonMACE study)

*ACR* albumin-creatinine ratio, *ADPKD* autosomal dominant polycystic kidney disease, *ALP* alkaline phosphatase, *BET* bromodomain and extraterminal, *CKD* chronic kidney disease, *CTGF* connective tissue growth factor *CVD* cardiovascular disease, *DKD* diabetic kidney disease, *eGFR* estimated glomerular filtration rate, *ESKD* end-stage kidney disease, *FSGS* focal segmental glomerulosclerosis, *IgAN* IgA nephropathy, *MACE* major adverse cardiovascular event, *TGF-β* transforming growth factor beta

#### Pirfenidone

TGF-β is a core regulator in fibrosis, and several TGF-β-neutralizing antibodies and small-molecule inhibitors have been developed for clinical trials. Fresolimumab and LY2382770, both anti-TGF-β antibodies, failed to show efficacy in steroid-resistant FSGS and DKD patients, respectively (NCT01665391, NCT01113801).^252^ As a small synthetic molecule, pirfenidone is protected against pulmonary fibrosis by the inhibition of TGF-β1 expression.^253,254^ What’s more, it is encouraging to find Pirfenidone could restore the eGFR in patients with DKD or FSGS.^255^ And more phase 2 and 3 trials are ongoing to study the effect of pirfenidone on CKD.

#### FG-3019

As a TGF-β downstream effector, CTGF could trigger fibroblast activation and ECM accumulation. In phase 1 clinical study, anti-CTGF antibody FG-3019 was well tolerated and led to a reduction of microalbuminuria in DKD patients.^256^ However, another phase 1 trial in primary FSGS was prematurely terminated. A phase 2 trial recruiting subjects with type 2 diabetes and persistent proteinuria was also terminated due to business purpose.

#### Bardoxolone methyl

Bardoxolone methyl, an NRF2 activator, was found to restore eGFR of different kidney diseases (e.g., DKD, IgAN, FSGS, Alport syndrome, and polycystic kidney disease) in a phase 2 trial. Several phase 2 trials reproduced the result that Bardoxolone methyl could increase the eGFR in CKD patients.^257,258^ However, in a larger phase 3 trial including more than 2000 patients with stage 4 CKD and diabetes (BEACON study), bardoxolone methyl failed to reduce the risk of ESRD or death from cardiovascular causes, along with an increased rate of cardiovascular events.^259^ Researchers conducted post-hoc analysis of BEACON study, and found that patients randomized to bardoxolone methyl were significantly less likely to experience the composite renal endpoint.^260^ Therefore, studies to further assess the potential risk-benefit profile of bardoxolone methyl are ongoing in patients without identified risk factors for fluid overload. These studies aim to ascertain whether increases in eGFR with bardoxolone methyl offer the potential to prevent or delay kidney function decline and progression to ESKD.

#### Apabetalone

Apabetalone is an oral inhibitor of BET proteins. Although in a large multicenter randomized controlled trial (BETonMACE study), apabetalone added to standard therapy did not significantly reduce the risk of major adverse cardiovascular events (MACE) among patients with recent acute coronary syndrome, type 2 diabetes, and low high-density lipoprotein cholesterol levels,^261^ a post-hoc analysis showed apabetalone demonstrated a potential cardioprotective effect in CKD patients with favorable effects on eGFR.^262^ Another post-hoc analysis of CKD subjects from the SUSTAIN and ASSURE randomized controlled trials also exhibit favorable effects of apabetalone on eGFR.^263^

#### Other drugs

RG-012, an inhibitor of miR-21, is currently evaluated in a Phase I trial for treating Alport syndrome. Galectin-3 regulates basic cellular functions such as cell-cell and cell-matrix interactions, growth, proliferation, differentiation, and inflammation. So, this protein is involved in fibrosis, chronic inflammation and scarring affecting many different tissues.^264^ Galectin-3 has been proposed as a novel biomarker of heart failure and cardiac fibrosis. And higher plasma galectin-3 levels were associated with an elevated risk of developing incident CKD.^265^ GCS-100 is a modified citrus pectin and galectin-3 antagonist. Clinical trials of different stages that study effect of GCS-100 on CKD are ongoing and promising results are expected.

## Conclusion and perspectives

The global prevalence of CKD has been a tremendous burden. Regardless of etiology, kidney fibrosis is a hallmark of most progressive CKD. After decades of work, the key steps in the understanding of kidney fibrosis have been unremittingly revealed: firstly, kidney damage-triggered inflammation activation and immune cell infiltration; secondly, profibrotic mediator release such as growth factors, chemokines, and cytokines; thirdly, myofibroblast activation and excessive ECM accumulation in tubulointerstitium, owing to ECM synthesis/degradation imbalance; fourthly, phenotype alteration and irreversible loss of parenchymal cells; fifthly, kidney microvasculature reduction. Detailed molecular mechanisms have been uncovered gradually. A recent report of human kidney atlas identified these primary cell types in kidneys, recapitulated several known subtypes, and clarified their key functions, guiding kidney disease classification when intricate pathophysiologic mechanisms underlie convergently clinical symptoms. Important findings also included the fact that PT S3 segment and medullary thick ascending limb (mTAL) were the two most vulnerable sites to kidney injury.^11^ Now, targeting the main collagen-producing myofibroblast activation is a promising strategy to prevent tubule damage and exert antifibrotic activity. However, no effective drugs exist at present and most of therapies only retard the progression of fibrotic kidney disease, underscoring the urgent need to explore innovative approaches to reverse or stop disease progression.

For a long time, we studied the mechanism of fibrotic kidney using whole-kidney tissue. However, this in reality may only represent changes in the renal tubular cells, as the tubules make up the majority of the kidney. Following the advancement of single-cell technology, many key questions have been answered, such as what kind of renal tubules are profibrotic, where myofibroblasts originate, which immune cells get involved, and how cells communicate with each other. Genetics and epigenetics are deeper mechanisms that regulate renal fibrosis. Although the reversible feature of epigenetics provides a fighting chance to treat the disease, epigenetic drugs are approximately restricted in the oncology field and few pioneering studies explore epigenetic clinical significance in kidney disease. However, genomic and epigenomic analyses offer ponderable diagnostic or prognostic biomarkers in the progression of kidney disease.

While exploring approaches against kidney fibrosis, investigators have sought to use drug delivery systems to target specific cells (PT cells, mesangial cells, myofibroblasts, et al.) for inhibiting myofibroblast activity and ECM production, simultaneously avoiding drug toxicity. Extracellular vesicles (EVs) are kinds of lipid bilayer-delimited particles secreted by almost all types of cells in physiopathologic conditions.^266^ EVs-contained mRNAs, miRNAs, proteins, and lipids from parent cells could be functionally transferred to recipient cells, and these messages mediate cell-to-cell communication. EVs are not only mediators of paracrine secretion, they have been applied as a drug delivery system for kidney diseases.^267,268^ Both traditional and biological drug delivery systems have demonstrated good safety and therapeutic efficacy in preclinical studies, but investigators appear to be very cautious about conducting clinical trials with them.

Overall, it is exciting to find more and more existing drugs, e.g., RAS blockage, SGLT2 inhibitors, vasopressin receptor 2 antagonist, and non-steroidal anti-mineralocorticoid can delay the progression of CKD. Many Chinese herbal formulas, single herbs, and Chinese herbal compounds have been shown to reduce kidney fibrosis.^269,270^ Solid evidence from well-designed experimental studies has revealed their antifibrotic mechanism and activity.^271,272^ However, challenges and barriers of traditional Chinese medicines still limited their participation in clinical trials for the treatment of kidney disease. The side-effect from traditional Chinese medicine is drastically under-reported. There is still lack of long-term follow-up data for some patients taking traditional Chinese medicine. Most of clinical investigations referred to traditional Chinses medicines against kidney diseases have issues such as flawed methodologic approach, suboptimal reporting, and small-sample size, etc.^269^

Kidney fibrosis is the common outcome across all kinds of progressive CKD, which means that kidney injury is in an advanced stage. This may be the reason why antifibrotic treatment is difficult. The prerequisite for early treatment is early detection. However, there is a lack of good biomarkers to predict and assess kidney fibrosis in clinic. There is no doubt that technological advances drive a deeper understanding of diseases. With a fuller understanding of fibrotic kidney mechanism, access to drugs stopping or even reversing renal fibrosis is challenging and promising.
